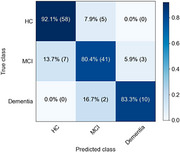# CognoSpeak: An automatic, remote assessment of early cognitive decline using speech

**DOI:** 10.1002/alz70856_102659

**Published:** 2025-12-26

**Authors:** Madhurananda Pahar, Fuxiang Tao, Bahman Mirheidari, Rebecca Bright, Swapnil Gadgil, Caitlin H Illingworth, Dorota Anna Braun, Lise Sproson, Ronan O'Malley, Heidi Christensen, Daniel J. Blackburn

**Affiliations:** ^1^ University of Sheffield, Sheffield, South Yorkshire, United Kingdom; ^2^ University of Sheffield, SHEFFIELD, United Kingdom; ^3^ Therapy Box, London, United Kingdom; ^4^ NIHR Health Research Centre in Long Term Conditions ‐ Devices for Dignity for Dignity, Sheffield, United Kingdom; ^5^ University of Sheffield, Sheffield Teaching Hospitals, Sheffield, United Kingdom

## Abstract

**Background:**

The benefits of detecting early signs of dementia include timely treatment and access to support. This can be challenging as it requires referral from primary care to secondary care for thorough investigations work‐up. There are long waits for secondary care assessment and lack of sufficient skilled staff to do this. Therefore, there is an a need for remote, smart technologies to support healthcare services to deliver timely and accurate diagnosis.

**Methods:**

CognoSpeak collects speech audio and video recordings when a virtual agent prompts the subject to answer a diverse range of questions on a web‐based app. The subjects' cognitive status was grouped into three groups: dementia, MCI, and healthy controls (HC). We have recruited over 1000 Healthy volunteers and >200 people with MCI and dementia. <3% have a negative view of the system or the 4 virtual clinicians. We have qualitative interview results from HV, GPs and people with MCI being presented at other abstracts at AAIC 2025.

**Results:**

We have results from 126 subjects (8 with dementia, 39 with MCI and 79 HC. Classification accuracy is shown in Figure 1

**Conclusion:**

CognoSpeak is a low‐cost, repeatable, non‐invasive tool that shows promise for use in clinical practice and in clinical trials as it can be done quickly, from home. It can also track mood and anxiety. We will present results from a larger data set for the AAIC conference in July, including accuracy of predicting mood and anxiety as well as cognition. We will also present a more detailed analysis of semantic and phonemic fluency scores